# A Potent Burkholderia Endophyte against Boxwood Blight Caused by *Calonectria pseudonaviculata*

**DOI:** 10.3390/microorganisms8020310

**Published:** 2020-02-24

**Authors:** Ping Kong, Chuanxue Hong

**Affiliations:** Virginia Tech, Hampton Roads Agricultural Research and Extension Center, 1444 Diamond Springs Road, Virginia Beach, VA 23455, USA; chhong2@vt.edu

**Keywords:** biocontrol, box blight, *Cylindrocladium buxicola*, *Cylindrocladium pseudonaviculata*, plant biosecurity, sustainable disease management

## Abstract

*Calonectria pseudonaviculata* (*Cps*) poses an increasing threat to boxwood, a major nursery crop and iconic landscape plant worldwide. Here, we report on a potent biocontrol agent that produces small sage green (SSG) colonies on potato dextrose agar. SSG is a bacterial strain recovered from Justin Brouwers boxwood leaves with unusual response to *Cps* inoculation. Water-soaked symptoms developed on leaves 2 days after inoculation then disappeared a few days later. This endophyte affected several major steps of the boxwood blight disease cycle. SSG at 10^7^ cfu/mL lysed all conidia in mixed broth culture. SSG at 10^8^ cfu/mL reduced blight incidence by >98% when applied one day before or 3 h after boxwood were inoculated with *Cps*. Its control efficacy decreased with decreasing bacterial concentration to 10^3^ cfu/mL and increasing lead time up to 20 days. When applied on diseased leaf litter under boxwood plants, SSG reduced *Cps* sporulation and consequently mitigated blight incidence by 90%. SSG was identified as a new member of the *Burkholderia cepacia* complex with distinct characters from known clinical strains. With these protective, curative, and sanitizing properties, this Burkholderia endophyte offers great promise for sustainable blight management at production and in the landscape.

## 1. Introduction

Boxwood blight caused by *Calonectria pseudonaviculata* (*Cps*) is an economically and ecologically important disease [1,2]. This disease was first reported in New Zealand [3] and the United Kingdom [4]. In the United States, boxwood blight was first confirmed in Connecticut and North Carolina [5], and now it has been reported in 28 states [1,6]. *Cps* attacks *Buxus*, a genus of slow-growing evergreen shrubs including English boxwood (*Buxus sempervirens* ‘Suffruticosa’), an iconic landscape plant in many public and private gardens [7,8]. In fact, English boxwood is one of the most susceptible boxwood cultivars evaluated to date, and may be destroyed by *Cps* within a week of inoculation [9,10,11]. This pathogen also attacks several species of pachysandra [12,13,14,15], sweet box [16,17,18], and potentially some common groundcovers and boxwood companion plants outside of the Buxaceae family [19].

Boxwood blight has rampaged in Europe [20] and now in North America due to limited control options [1]. First, no boxwood cultivar is immune to the blight disease although some cultivars are more tolerant than others [21,22,23,24,25]. Second, chemical protection, although effective [26,27], is costly due to high fungicide prices, a long season of susceptibility (7 months or more), short retreatment intervals needed for effective control, and the required labor. Fungicide protection also presents significant hazards to human and environmental health. Specifically, chlorothalonil, one of the most effective compounds, has recently been classified as a category one carcinogen and removed from the market in Europe [28]. In addition, use of fungicides in historic gardens and residential and commercial landscapes is particularly challenging. Third, mulching may prevent soil inoculum from splashing with water onto plant foliage, but it cannot protect boxwood from other sources of inoculum or other avenues of pathogen dispersal [29].

In search for safer, more effective and sustainable disease control methods, several recent studies have used *Buxus sempervirens* ‘Justin Brouwers’, a highly susceptible cultivar, to explore the potential of biological control for blight mitigation. Among the biofungicides evaluated to date, RootShield Plus reduced blight incidence by 44% under high inoculum loads and conducive environments [30]. *Trichoderma koningiopsis* Mb2, an isolate recovered from a collapsing wild mushroom, reduced disease incidence by 54% to 63% under similar high disease pressure environments [31]. Likewise, *Pseudomonas protegens* 14D5, a strain isolated from nursery recycled irrigation water, reduced blight incidence by >50% [32]. More recently, *Pseudomonas lactis*, an endophyte isolated from boxwood leaves, provided up to 72% blight control depending upon the pretreatment lead time [33].

Endophytes are microorganisms that reside within various tissues of a host plant in a commensal or beneficial manner [34]. These microorganisms have received considerable attention lately due to their potential for crop disease control [35,36,37,38], plant growth, and sustainable agricultural productivity [39]. Here, we report on a potent boxwood endophyte, *Burkholderia* sp., against *Cps*. Specifically, we assessed (1) how this bacterial endophyte and its metabolites may affect *Cps* survival, (2) how it may protect susceptible plants from infection by *Cps*, and (3) how this endophyte may prevent fallen diseased leaves from becoming a source of inoculum for new infection. Additionally, we sequenced its 16S ribosomal RNA and *RecA* genes, analyzed the restriction fragment length polymorphism of *RecA*, tested it against the *Burkholderia cepacia* Epidemic Strain Marker and its responses in the onion maceration assay [40,41,42,43,44] in order to determine its species identity and differentiate it from clinical strains in the *B. cepacia* complex (Bcc).

## 2. Materials and Methods

### 2.1. Cps Isolate and Conidia Production

A *Cps* isolate, 12A01 was used in this study. This isolate, although recovered from affected sweet box (*Sarcococca hookeriana* var. *humilis*), was part of the same clone introduced to a private garden via infected English boxwood [16] and has the same virulence as those from boxwood on the same garden as shown in a comparative study [9]. Cultures were grown and maintained at 25 °C on Difco™ potato dextrose agar (PDA, Becton, Dickinson and Company, Sparks, MD, USA). Conidia were produced using fresh potato dextrose broth (PDB) as described by [31]. Briefly, a small amount of mycelia was scraped from the culture surface then evenly distributed in 90 mm plates with fresh PDB. After a 4 day incubation at 25 °C, nutrient medium was decanted. Mycelial mats that formed and were attached to the plate bottom were rinsed with sterilized distilled water (SDW). These washed plates were then placed under fluorescent light at 1200 lux to induce production of conidia. Conidia were harvested with SDW or 0.01% Tween™ 20 (Croda Inc., New York, NY, USA) into a beaker; spore concentration was determined with a hemocytometer.

### 2.2. Plant Growth Conditions and Biosafety Measures for All in-Planta Inoculation Studies Buxus Sempervirens

Justin Brouwers boxwood was used in all inoculation studies. Blight-free liners were donated by Saunders Brothers Inc. (Piney River, VA, USA). Two liners were potted in a 15 cm plastic pot with pine bark-based potting mix and fertilized once with slow release fertilizer (Harrell’s LLC, Lakeland, FL, USA). Plants were irrigated up to three times a day, depending upon the time of year, and maintained on a gravel pad until use at the Virginia Tech Hampton Roads Agricultural Research and Extension Center in Virginia Beach, VA, USA.

To prevent *Cps* from spreading to nearby boxwood plantings, all inoculation studies were done in a laboratory with restricted access. During each experiment, plants were placed in Ultra™ Latching Storage Boxes (66 cm × 41 cm × 50 cm, Sterilite Corporation, Townsend, MA, USA) to further contain the diseased plant materials. At the termination of experiments, all used plants and planting materials were autoclaved before disposal, while used boxes and other tools are washed and decontaminated with 70% ethanol.

### 2.3. Isolation and Selection of the Bacterial Endophyte from Boxwood Leaves with Unusual Response to Cps Inoculation

Detached leaves of Justin Brouwers boxwood were washed in tap water then surface sterilized with 70% ethanol and inoculated with a drop of *Cps* conidia. Water-soaked lesions developed on individual leaves within 2 days. However, not all of these lesions progressed further; instead, some disappeared at 7 days post inoculation (Figure S1).

The leaves showing symptom reversion were surface sterilized again with 70% ethanol, cut into small pieces, then vortexed in SDW for 10 min. When the leaf debris settled to the bottom, 100 µL of the supernatant was plated onto PDA then incubated at 25 °C for 48 h. Resultant bacterial colonies were grouped by color and size; eight representative colonies were subcultured on Difco nutrient agar (NA, Becton, Dickinson and Company, Sparks, MD, USA) for initial evaluation using a dual culture assay. Briefly, a mycelial plug of *Cps* was placed in the center of 90 mm PDA plates and a bacterial isolate from a 24 h liquid culture in Difco nutrient broth (NB, Becton, Dickinson and Company, Sparks, MD, USA) was streaked equidistantly on its left and right sides. Control plates were streaked with NB without small sage green (SSG). The assay was done three times with slightly different timings of *Cps* seeding in relation to bacterial streaking: 3 days before in the first run, at the same time in the second run, and 16 h later in the third run. Each run included three replicate plates per bacterial isolate. All plates were incubated at 25 °C in the dark. The diameter of the *Cps* colony in each plate was measured 4 weeks later. The bacterial isolate that produced small sage green (SSG) colonies on PDA was consistently most effective against *Cps* growth (Figure S2); subsequently, it was selected for further evaluation.

### 2.4. SSG Cell Suspension and Cell-Free Supernatant Preparation

SSG was maintained on PDA plates. For liquid culture, a 4 mL NB was inoculated with a single colony and incubated on a G24 Environmental Incubator Shaker (New Brunswick Scientific Inc., Edison, NJ, USA) at 180 rpm and 28 °C overnight, then used as a culture stock. For experiments, 150 mL NB or PDB was inoculated with 1 mL of the SSG stock then incubated for 40 h under the same conditions. The culture was centrifuged at 14,210× *g* for 15 min. Bacterial cells in pellets were resuspended in 200 mL PDB for in vitro assays or 0.01% Tween 20 for plant inoculation studies. Bacterial cell concentration of resultant resuspensions was determined by spreading 100 µL of its dilutions on PDA then counting the emerging colonies after a 2 day incubation at 25 °C. The resultant bacterial cell concentrations ranged from 10^8^ to 10^9^ colony-forming units (cfu) per milliliter. In the meanwhile, supernatant was further passed through a 0.22 μm filter to produce cell-free supernatant (CFS). Resuspended bacterial cells and CFS were evaluated separately for their potential against *Cps* unless stated otherwise. The resuspended bacterial cells and CFS treatments hereafter were referred to as Cell and CFS, respectively.

### 2.5. SSG Effect on Cps Conidia Survival and Germination

Three treatments, Cell at 10^7^ cfu/mL, CFS, and PDB only as a control, were included in this study. A 100 µL aliquot of *Cps* suspension at 10^4^ conidia/mL SDW was mixed with 700 µL of Cell, CFS, or PDB in Costar™ 24 well Flat Bottom Cell Culture Plates (Corning Inc., Corning, NY, USA). The mixtures were incubated at 25 °C in the dark for 1, 4, 8, 24, or 48 h then examined for conidia lysis, germination, and germling differentiation using an Olympus IX71 inverted microscope (Olympus Corporation of the Americas Headquarters, Center Valley, PA, USA) at magnification of 100×. This assay included triplicate wells per treatment and was done twice.

### 2.6. Effect of SSG on Boxwood Blight

Four treatments were included in this study: (1) Cell at 10^8^ cfu/mL 0.01% Tween 20, (2) CFS, (3) 0.01% Tween 20 (as the control for the Cell treatment), and (4) NB (as the control for CFS). Boxwood foliage was pretreated at 20 mL/plant using hand sprayers one day prior to being challenged with *Cps*. This experiment had triplicate plants per treatment and was done three times. Treated plants were arranged in a randomized complete block design in the storage boxes for inoculation with *Cps* at 1 to 5 × 10^4^ conidia/mL 0.01% Tween 20 at 20 mL/pot. Inoculated plants were kept in closed storage boxes for 48 h to facilitate infection. Diseased leaves and healthy-looking leaves on each plant were counted 7 days post inoculation. Disease incidence was calculated by dividing the diseased leaf count by the total leaf count.

### 2.7. Effect of SSG Concentration on Boxwood Blight

Five concentrations of 0, 10^3^, 10^5^, 10^7^ and 10^9^ cfu/ml 0.01% Tween 20 were included in the initial run. Based on the results from the initial run, the concentration of 10^3^ cfu/mL was excluded from the second run. Plant pretreatment including lead time of one day, Cps inoculation, and disease assessment all were performed as described above with two minor changes. Cps inoculum concentration was at 5 × 10^4^ and 2 × 10^4^ conidia/mL in the initial and repeated runs, respectively. Likewise, the highest SSG cell concentration was slightly greater in the first than the second runs (3 × 10^9^ vs. 2 × 10^9^ cfu/mL).

### 2.8. Effect of SSG Treatment Lead Time on Boxwood Blight

All boxwood plants were treated with SSG at 4 × 10^8^ cfu/mL or 0.01% Tween 20 as control. A quarter of the plants pretreated with SSG and those with Tween 20 were inoculated with *Cps* at 2 × 10^4^ conidia/mL 1, 10, 20, and 30 days later. SSG pretreatment, *Cps* inoculation, and disease assessment were all performed as described above for the SSG concentration experiment. Blight control was calculated for each lead time by dividing the difference in disease incidence between SSG treated and control plants by that of the control plants. This experiment was repeated once with SSG at 2 × 10^9^ cells/mL and *Cps* at 10^4^ conidia/mL.

### 2.9. Effect of Post-Inoculation SSG Treatment on Boxwood Blight

This study included application of resuspended SSG cells at three time points: 3, 24, and 48 h post inoculation, plus a nontreated control. *Cps* inoculation, SSG treatment, disease assessment, and blight control calculations were done as described above for the lead-time experiment. The experiment was conducted twice with SSG at 5 × 10^8^ cfu/mL and 4 × 10^8^ cfu/mL while *Cps* was at 5 × 10^4^ conidia/mL and 2 × 10^4^ conidia/mL in the first and second runs, respectively.

### 2.10. SSG Effect on Potential of Diseased Leaves as a Source of Inoculum

This study began with collecting and air-drying diseased leaves then storing them in a cold room at 4 °C for 15 months. One hundred stored leaves were spread on the surface of potting mix under healthy Justin Brouwers plants in a container, then immediately cover sprayed (not onto the boxwood foliage) with a mixture of 35 mL NB culture of SSG and 35 mL SDW at a final concentration of 10^8^ cfu/mL or ½ strength NB without SSG. Each treatment included three replicate plant containers and treatments were arranged in a randomized complete block design. After 24 h, plants in treated containers were overhead watered using a watering can, which was repeated every other day until the end of the experiment. To prevent cross contamination between treatments through movement of accumulated water at the bottom of boxes, plant containers were placed on inverted empty containers. Three sets of data were collected with *Cps* sporulation on leaf litter and blight incidence on boxwood foliage assessed six times while, SSG survival in the potting mix was determined twice post treatment. This experiment was conducted twice.

*Cps* ability to sporulate on control and SSG-treated leaf litter was assessed at 5, 10, 20, 30, 40, and 50 days post treatment. On each assessment day, ten leaves were collected from each pot and placed onto mesh overlaid moist paper towels in closed plastic crispers for 5 days. These leaves were placed in a test tube with 10 mL 0.01% Tween 20 then vortexed for 10 min to dislodge conidia. Concentrations of conidia in resultant suspension were determined with a hemocytometer, and six independent counts were averaged for each replicate sample. The per ml conidia concentration was equivalent to the number of conidia produced per leaf. This number was then divided by Justin Brouwers average leaf size of 2 cm^2^ to calculate the number of conidia produced per unit leaf area.

Blighted leaves including those fallen ones were counted for each plant. Total number of leaves on each plant was estimated by counting the number of branches then multiplying by a factor of 26 leaves per branch, which was predetermined based on the branch and leaf counts for plants in randomly selected 24 pots. Disease incidence was determined by dividing the diseased leaf count by total leaf estimate.

To determine SSG survival in soilless potting mix, a 100 mg sample was taken from the top 2 cm in each container using a straw. Each potting mix sample was added to a test tube with 10 mL SDW then vortexed for 15 min. After diluting 10 to 10^6^ times, 100 µL of original prep, or a dilution was spread onto a PDA plate after the debris settled. Small sage green colonies were counted after a 72 h incubation at 25 °C. Their SSG identity was verified with *Burkholderia cepacia* selective agar (BCSA, LabGenome, Houston, TX, USA).

### 2.11. SSG Species Identity and Differentiation from Epidemic Strains of Burkholderia cepacia

Three major steps were taken to determine SSG species identity. First, SSG was streaked onto BCSA medium and incubated at 25, 35, and 42 °C for 72 h [45] to determine whether it belongs to the Bcc. Second, DNA was extracted from SSG cells, then 16S rRNA and *RecA* genes were amplified by PCR using the universal primers 27F, 968F, and 1410R [40], and primers BCR1 and 2 [41], respectively. PCR products were sequenced at Eton Bioscience (Research Triangle Park, Raleigh, NC, USA). Processed sequences were deposited into GenBank (Accession: MK424809 for *RecA* gene and MK418913 for 16S DNA) and blasted against existing sequences in the repository (http://blast.ncbi.nlm.nih.gov) and at EzBioCloud [46] to determine the identity of this bacterial endophyte. Third, *RecA* PCR products were digested with *Hae*III and *Mnl*I then their RFLP were analyzed as described previously [41] to determine their genomovar association in the Bcc.

Two additional steps were taken to assess SSG risk as a human health hazard. First, bacterial DNA was amplified with specific primers for the Bcc epidemic strain marker (BCESM) [42] to determine whether this endophyte is associated with any known opportunistic human pathogens in the complex. Second, an onion maceration assay was conducted to differentiate SSG from clinical strains that generally do not macerate onion bulb scale tissue [43,44]. Briefly, pieces of fresh onion scales were wounded with a sterilized needle and inoculated with 10 µL of 40 h SSG culture at 10^8^ cells/mL or NB as the control. The inoculated onion scales were incubated at 25 °C in a moist container, and symptom development of onion scale tissue was recorded after 3 days.

### 2.12. Data Analysis

Data from different experimental runs, if homogenous, were pooled then subjected to analysis of variance (ANOVA) to determine the level of difference among treatments and that of interactions among factors using Statistical Analysis Software version 9.4 (SAS Institute, Cary, NC, USA). Otherwise, they were analyzed by experimental run. Treatment means were separated according to the least significant difference (LSD) test at *p* = 0.05.

## 3. Results

### 3.1. SSG Effect on Cps Conidia Survival and Germination

**Figure 1:**
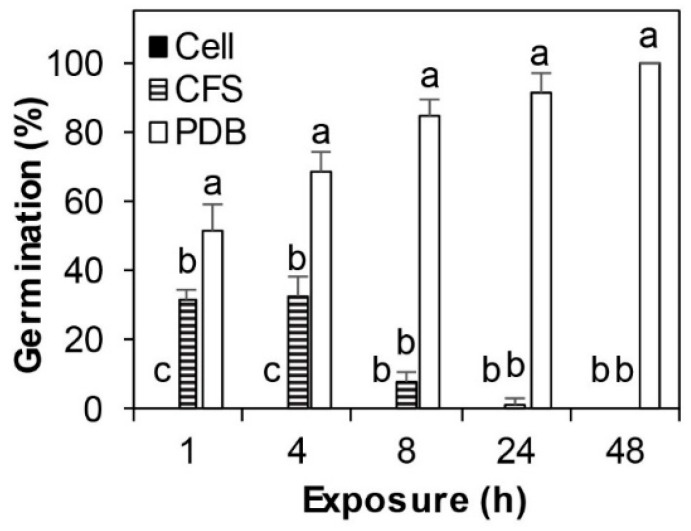
Conidia germination of *Calonectria pseudonaviculata* after being incubated in small sage green (SSG) cell suspension at 10^7^ cfu/mL (Cell), cell-free supernatant (CFS), or potato dextrose broth (PDB). Each column is an average of six replicates from two repeated assays and is topped by a standard error bar. Columns marked with the same letter within each exposure time did not differ according to the least significant difference (LSD) test at *p* = 0.05.

**Figure 2:**
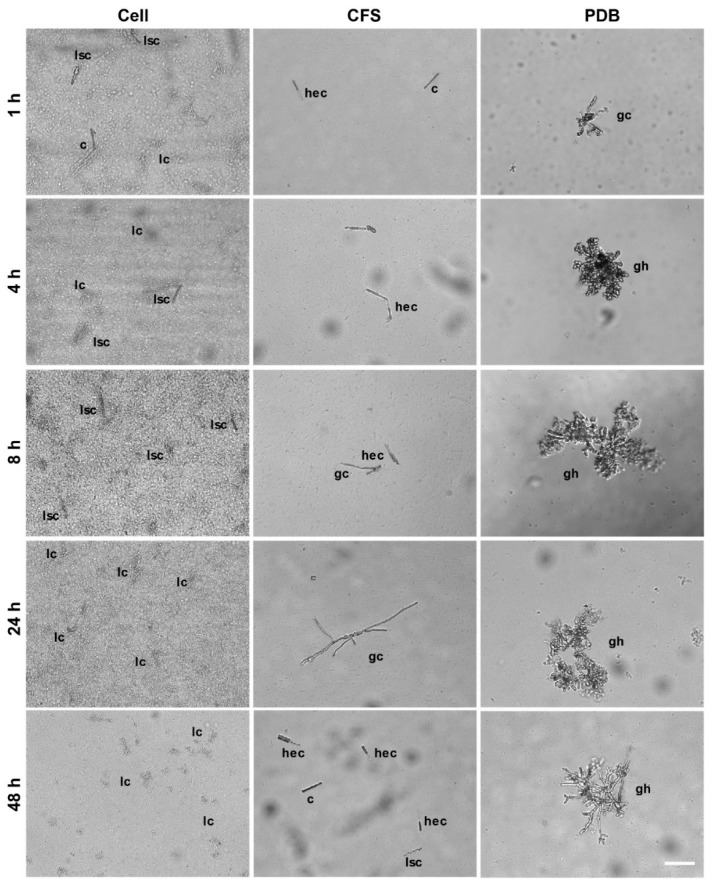
Micrographs of conidia morphology of *Calonectria pseudonaviculata* after being incubated in SSG cell suspension at 10^7^ cfu/mL (Cell), cell-free supernatant (CFS), or potato dextrose broth (PDB): c = conidium, lsc = lysing conidium, lc = lysed conidium, hec = half empty conidium, gc = germinating conidium, gh = growing hyphae from conidial germling. Bar = 50 µm.

Difference was observed in germination of conidia among Cell, CFS, and PDB treatments (*p* < 0.01), but not between the two experimental runs (*p* = 0.19) nor among the exposure times (*p* = 0.23). There were, however, significant interactions between treatment and exposure time (*p* < 0.01). About 52% conidia in control wells with PDB germinated within 1 h; their germination rate increased with time, reaching 100% at 48 h (Figure 1) with germling aggregation and further development into hyphae (Figure 2). In contrast, conidia germinated at a much lower a rate in wells with CFS than those in control wells. None of the conidia in wells with SSG cells germinated over the 48-h period; in fact, they were all lysed. Similar conidial lysis was observed in wells with CFS, but to a lesser extent. Conidia that had not been lysed had an empty cell, they germinated poorly, and fewer germlings developed further (Figure 2).

### 3.2. Effect of SSG on Boxwood Blight

Substantial blight control was seen on Justin Brouwers plants pretreated with resuspended SSG cells or CFS one day prior to inoculation with *Cps*. Resuspended SSG cells were consistently more effective than CFS for blight control in all experiments (*p* < 0.01). The former reduced blight incidence by nearly 100% when compared to the control with 0.01% Tween 20. Comparatively, CFS reduced blight incidence by 73% when compared to the control with NB. No difference in blight control among three experimental runs (*p* = 0.31) nor any interaction between experiment run and treatment (*p* = 0.35) was observed.

### 3.3. Effect of SSG Concentration on Boxwood Blight

Greater blight severity was observed (*p* < 0.01) on control plants in initial than repeated experiments (98% vs. 33% leaves blighted) due to the difference in *Cps* inoculum concentration between the two runs (5 × 10^4^ vs. 2 × 10^4^ conidia/mL).

**Figure 3:**
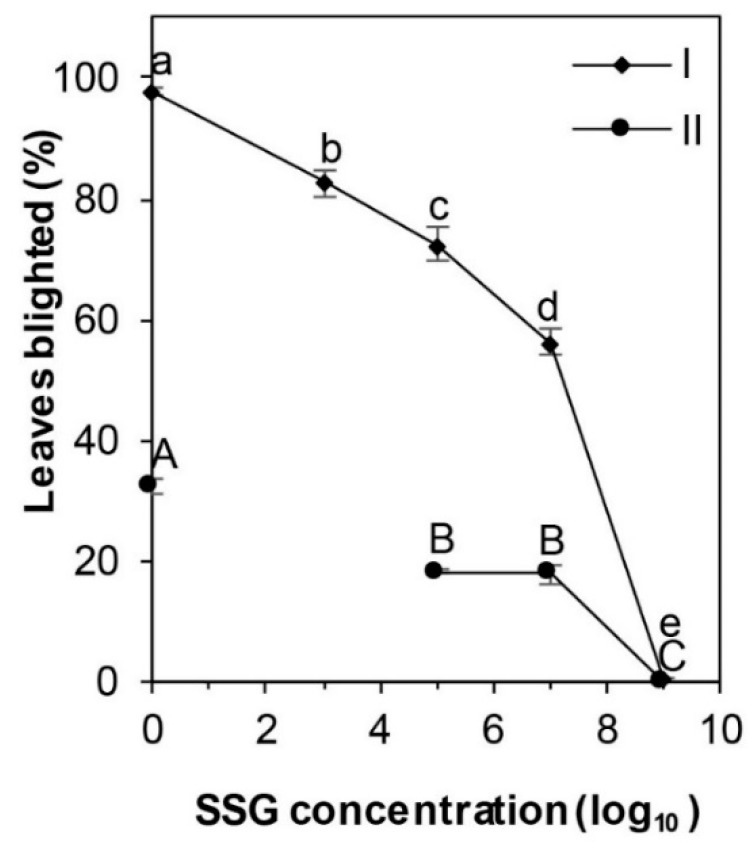
Leaf blight incidence on *Buxus sempervirens* ‘Justin Brouwers’ boxwood decreased with increasing SSG concentration from 0 to 10^9^ cfu/mL applied one day prior to inoculation with *Calonectria pseudonaviculata* at 5 × 10^4^ and 2 × 10^4^ conidia/mL in the initial (I) and repeated experiments (II), respectively. Boxwood blight was assessed 7 days after inoculation. Data points represent mean leaves blighted (%) of three replicate plants and are presented with a standard error bar. Means marked with the same small case letter did not differ in the first run and those with the same uppercase letter did not differ in the repeated run according to the LSD test at *p* = 0.05.

Blight incidence decreased (*p* < 0.01) with increasing SSG cell concentration: 83% on boxwood pretreated with SSG at 3 × 10^3^ cfu/mL and 1% on those at 3 × 10^9^ cfu/mL in the initial experiment (Figure 3). A similar decreasing trend was observed in the repeated experiment: 18% leaves blighted at 2 × 10^5^ cfu/mL and 1/10 percent at 2 × 10^9^ cfu/mL (Figure 3).

### 3.4. Effect of SSG Treatment Lead Time on Boxwood Blight

**Figure 4:**
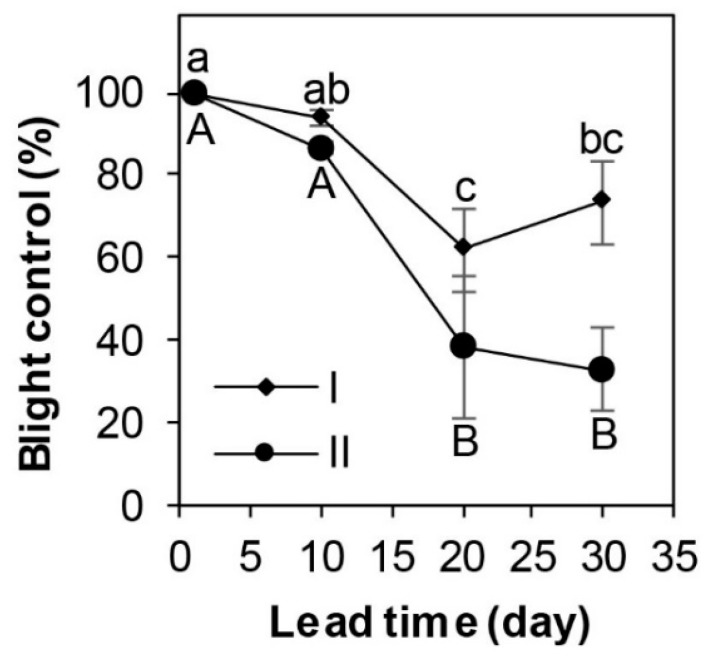
Blight control by SSG at 10^8^ cfu/mL decreased with increasing application lead time prior to inoculation with *Calonectria pseudonaviculata* at 1 to 2 × 10^4^ conidia/mL. Each data point is a mean blight control of three replicate Justin Brouwers boxwood plants and is presented with a standard error bar. Means marked with the same small case letter did not differ in the first run and those with the same uppercase letter did not differ in the repeated run according to the LSD test at *p* = 0.05.

When boxwood plants were cover sprayed with SSG at 4 × 10^8^ cfu/mL prior to inoculation with *Cps*, significant differences were observed in blight control between two experimental runs (*p* = 0.01) and four lead times (*p* < 0.01). Blight control was over 99% in both runs when SSG was applied one day prior to inoculation with *Cps*, and the control efficacy decreased with increasing lead time (Figure 4). Specifically, this decrease was significant between the lead times of 10 and 20 days, but not between 1 and 10 days, nor between 20 and 30 days in both runs.

### 3.5. Effect of Post-Inoculation SSG Treatment on Boxwood Blight

**Figure 5:**
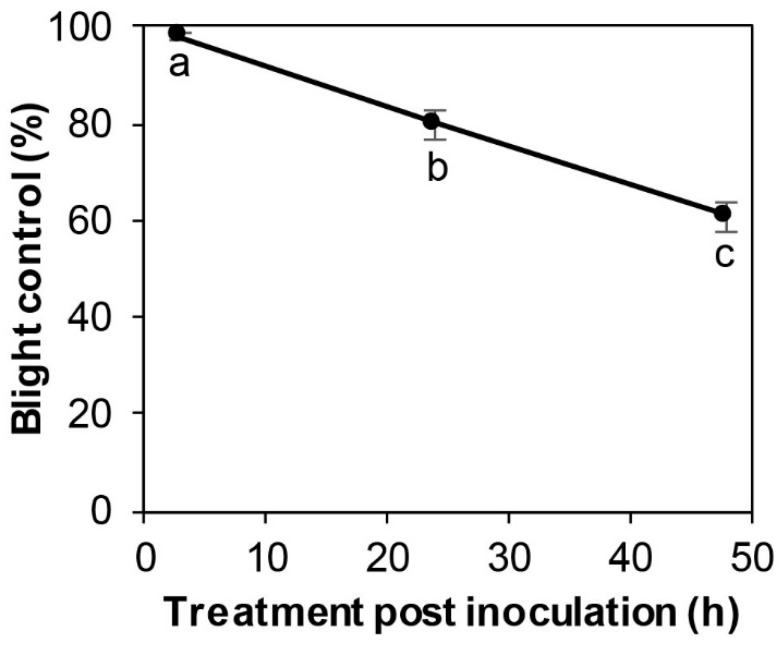
Blight control by SSG at 10^8^ cfu/mL decreased with increasing treatment time post inoculation with *Calonectria pseudonaviculata* at 10^4^ conidia/mL. Each data point is a mean blight control of six replicate Justin Brouwers boxwood plants from two experimental runs and is presented with a standard error bar. Data points marked with different letters differed according to the LSD test at *p* = 0.05.

SSG applied 3 h post inoculation reduced blight by 98% (Figure 5). However, blight control decreased (*p* < 0.01) with increasing time: 62% at 48 h post inoculation. No difference was observed in blight control between two experimental runs (*p* = 0.23).

### 3.6. SSG Effect on Potential of Diseased Leaves as a Source of Inoculum

**Figure 6:**
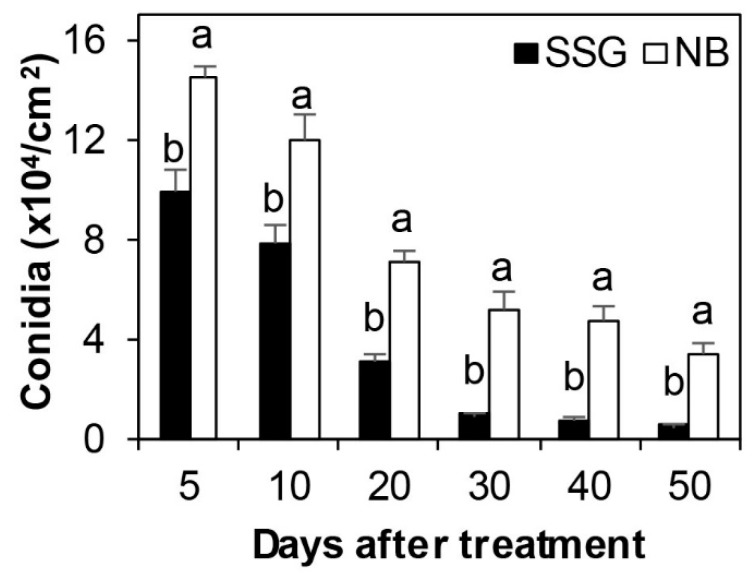
SSG reduced sporulation by *Calonectria pseudonaviculata* on blighted leaves for a range of time after they were placed under healthy plants then cover sprayed with SSG suspension at 10^8^ cells/mL (SSG) or with nutrient broth (NB) as a control. Each column depicts an average of six replicates from two experimental runs and is topped with a standard error bar. Columns marked with different letters within each sampling time differed according to the LSD test at *p* = 0.05.

More conidia were produced consistently on diseased leaf litter cover sprayed with NB than that treated with NB culture containing both SSG cells and metabolites in experiments (*p* < 0.01, Figure 6). Conidia production of both treatments decreased steadily with increasing post-treatment time (*p* < 0.01). The number of conidia produced on NB-treated leaf litter was 144,542/cm^2^ and 33,888/cm^2^ at 5 and 50 days post treatment, respectively. Likewise, the number of conidia produced on SSG-treated leaf litter was 98,756/cm^2^ and 5,231/cm^2^ at 5 and 50 days post treatment, respectively.

Conidia produced on the leaf litter resulted in boxwood blight on the lower portion of Justin Brouwers plants. However, a higher percentage of leaves developed blight symptoms from the control litter cover sprayed with NB than those treated with SSG (11% vs. 1%, *p* < 0.01). Difference was not observed in blight incidence between two experimental runs (*p* = 0.25) nor among six assessment dates (*p* = 0.70).

SSG population in sampled potting mix declined over the 50 day period (*p* < 0.01) with 6 × 10^9^ and 2 × 10^6^ cfu/g at the beginning and termination of the study, respectively. The variety and population of other microbes recovered along SSG also declined during the same period, and this decline was most obvious for fungi. SSG was never detected in the control potting mix treated with NB.

### 3.7. SSG Species Identity and Differentiation from Epidemic Strains of Burkholderia cepacia

Several lines of evidence supported SSG’s membership in the Bcc, although it does not seem to belong to any known species. It grew well in *B. cepacia* selective agar at 25 °C and 37 °C, but not 42 °C. Its 16S rDNA sequence also was 99% identical to many known species in the Bcc with *B. cepacia* JCM 2799 (Figure S3) and *Burkholderia* sp. JJOA-S as the closest match when being blasted through NCBI and EzBioCloud, respectively. Likewise, its *RecA* gene sequence had the best alignment with that of *B. cepacia* strain IST431 (Figure S4). When SSG *RecA* PCR products were digested with *Hae*III and *Mnl*I restriction enzymes, they produced a RFLP pattern that was similar to type “H” of genomovar III and type “d” of genomovar I, respectively. This result indicated that SSG belonged to neither genomovar I nor III because the expected RFLP pattern for genomovar I was “D” with *Hae*III digestion and for genomovar III was “g” with *Mn*/I digestion [41].

Both ID and RFLP indicated that SSG was distinct from all known clinical strains in the Bcc. In addition, SSG DNA was not amplified with the *B. cepacia* epidemic strain marker. Furthermore, unlike clinical strains, SSG macerated onion scale tissues (Figure S5).

## 4. Discussion

This study identified a potent *Burkholderia* endophyte, SSG, with both protective and curative properties against boxwood blight. As a protectant, SSG at 10^8^ cfu/mL applied one day prior to inoculation with *Cps* provided almost complete protection of Justin Brouwers boxwood, a highly susceptible cultivar under high disease pressure environments (Figure 4). This blight control efficacy is comparable to those of the most effective fungicides [10,27,47,48,49]. In fact, it performed much better than the most effective biofungicides and all biocontrol agents identified to date, including RootShield *Plus* [30], non-indigenous isolates of *Trichoderma koningiopsis* [31], and *Pseudomonas protegens* [32], as well as an indigenous strain of *Pseudomonas lactis* [33]. As a curative agent, SSG reduced blight by 98% when its cell suspension at 10^8^ cfu/mL was applied 3 h after inoculation with *Cps* (Figure 5).

In addition to directly protecting boxwood plants from infection by *Cps*, SSG greatly affected other major steps in the boxwood blight disease cycle. First, fallen diseased leaves are important sources of inoculum for new infection [29]. SSG reduced the pathogen’s ability to produce conidia on diseased leaf litter by 32% at 5 days after cover spray treatment; this reduction rate increased with time: 85% at 40 days and longer (Figure 6). Second, conidia are the major dispersal and infective structure for boxwood blight [21,22,50]. SSG at 10^7^ cfu/mL completely lysed conidia while CFS had a similar but lesser impact (Figure 1 and Figure 2). Together, SSG is an effective biological sanitizer and may be used to reduce the pathogen population at sites of infestation.

SSG may involve complex modes of action against *Cps*. First, some environmental Bcc species are known to produce unique antifungal antibiotics such as pyrrolnitrin, occidiofungin, and glidobactins [51]. SSG may produce these antibiotics for suppression of *Cps* mycelial growth although their species and mechanisms are yet to be elucidated. Second, SSG may also produce chitinases and glucanases that break down the cell wall building blocks including chitin, glucans, and other polymers during the lysis process. The fact that conidia were lysed at a much greater rate in wells with live bacterial cells than those with CFS supports this hypothesis. Further studies on the molecular interactions between SSG and *Cps* are warranted to understand the modes of action by which SSG acts against *Cps*. It is worth noting that when *Cps* and SSG were grown in different sections of multisection Petri plates, no suppression was observed. Thus, involvement of volatile compounds by SSG in *Cps* suppression is unlikely.

Many endophytes can elicit plant resistance during plant infection [35,52], and more than likely, SSG is able to do the same in boxwood against boxwood blight. This hypothesis is supported by the fact that boxwood plants remained moderately protected from infection by *Cps* even 20 days and longer after SSG treatment (Figure 4). SSG may have elicited plant defenses that provide persistent resistance to the disease although at low to moderate levels. Bcc bacteria survive poorly on leaf surfaces [53]. The sustained blight control by SSG in the present study may also be due in part to their entry into plant tissue at a low rate that may continually act against *Cps*. Further study on the interaction between plant and SSG is warranted to elucidate the mechanisms underlying the sustained blight control.

Bcc is a very diverse Burkholderia group including some opportunistic human pathogens that are associated with lung infection in patients with cystic fibrosis [51]. Although SSG belongs to Bcc, its profiles do not match with those of any known species in this complex and its likelihood of being a human pathogen is low. First, SSG was tested negative for BCESM, the standard human virulence marker used by the United States Environmental Protection Agency for registration of biocontrol products with Bcc as an active ingredient [42,54]. Second, SSG macerated fresh onion scales, a phenomenon not observed for known clinical strains [43,44]. Additionally, SSG was isolated from surface sterilized plant tissue whereas all clinical strains were isolated from human tissue or waste.

More investigations are warranted to clear SSG from any risk as a human pathogen. Though Bcc species and strains are active ingredients of several labeled biopesticides including Deny^®^, Blue Circle^®^, and Intercept^®^ [54], clearing SSG’s human health risk should be a priority in the further development of this potent biocontrol agent. This may be accomplished through experiments with animal models or identification of specific genes/markers that can differentiate pathogens from non-pathogens in the Bcc, and/or genome sequencing and annotation. Alternatively, further development may be focused on SSG metabolites for immediate applications.

Further to clearance on its human health risk, several technical questions must be answered in the future development of this biocontrol agent into a final product. All in-planta studies presented here were done under controlled environments in the lab. Thus, the first question of critical importance is how SSG may perform against boxwood blight under both production and landscape settings, preferably in multiple locations and climatic environments. Although SSG may protect boxwood from infection by *Cps* for 3 to 4 weeks, longer than most fungicides currently labeled for protecting boxwood and/or controlling Cylindrocladium diseases [27,30,48,49], its efficacy decreased with time (Figure 4). This reduction in SSG efficacy with time may be due in part to a declining bacterial population. Therefore, the second question of practical importance is how SSG interacts with boxwood and how its survival inside and on the surface of boxwood foliage may be improved for sustained high blight control performance. Other questions of significance are how SSG interacts with *Cps* and what are the mechanisms by which this endophyte acts against *Cps* and/or protects boxwood? Formulation and delivery method could have tremendous impacts on the performance of microbial biocontrol agents [55]. So, an additional question is how SSG may be formulated and delivered to enhance its entry and survival inside and on the surface of boxwood plants for sustained survival and blight control performance. Nevertheless, this study identified a highly potent biocontrol agent with protectant, curative, and sanitizing properties.

## 5. Conclusions

This study identified a Burkholderia endophyte, SSG, with strong protective, curative and sanitizing activities against the boxwood blight pathogen, *Calonectria pseudonaviculata* on containerized Justin Brouwers boxwood plants under controlled environments. This bacterial strain was identified as a new member of the *B. cepacia* complex which is distinct from all known clinical strains. Further investigations are warranted to further elucidate its interactions with the pathogen and boxwood plants, and clear the risk as a human pathogen.

## Supplementary Materials

The following figures are available online at https://www.mdpi.com/2076-2607/8/2/310/s1. Figure S1: Changes at inoculation sites on detached boxwood leaves after receiving a drop containing conidia of *Calonectria pseudonaviculata*. Top. Water-soaked lesion appeared on individual leaves 2 days after inoculation (dpi). Bottom. Water-soaked lesion disappeared from three leaves (in rectangle) while progressing to show blight symptoms and signs on other leaves at 7 dpi. Figure S2: Reduction in colony diameter of 4 week old *Calonectria pseudonaviculata* (*Cps*) by SSG and other boxwood endophytes coded by size (s = small, m = medium, l = large) and color (p = pink, sg = sage green, w = white, y = yellow), plus a number if more than one isolate of similar color types and sizes assessed in three dual culture assays with slightly different *Cps* seeding timings in relation to bacterial streaking: 3 days before (left), at the same time (middle), and 16 h later (right). Each column represents a mean of triplicate plates, topped with a standard error bar. Columns topped with the same letter within each assay did not differ according to the LSD test at *p* = 0.05. Figure S3: Neighbor-joining tree of SSG (query in yellow) and closely related *Burkholderia* strains in GenBank according to BLAST pairwise alignments of 16S rDNA. Figure S4: Neighbor-joining tree of SSG (query in yellow) and closely related *Burkholderia* strains in GenBank according to BLAST pairwise alignments of RecA gene. Figure S5: Symptoms on onion scales at 3 days after inoculation with a 10 µL drop of culture of SSG at 10^8^ cfu/mL or nutrient broth (NB) control.
