# Supplementary material for: A Potent Burkholderia Endophyte against Boxwood Blight Caused by Calonectria pseudonaviculata
Source: Microorganisms. 2020 Feb 24;8(2):310. doi: 10.3390/microorganisms8020310 (PMC7074863; doi:10.3390/microorganisms8020310)

Figure S1: Changes at inoculation sites on detached boxwood leaves after receiving a drop containing conidia of *Calonectria pseudonaviculata*. Top. Water-soaked lesion appeared on individual leaves 2 days after inoculation (dpi). Bottom. Water-soaked lesion disappeared from three leaves (in rectangle) while progressing to show blight symptoms and signs on other leaves at 7 dpi.

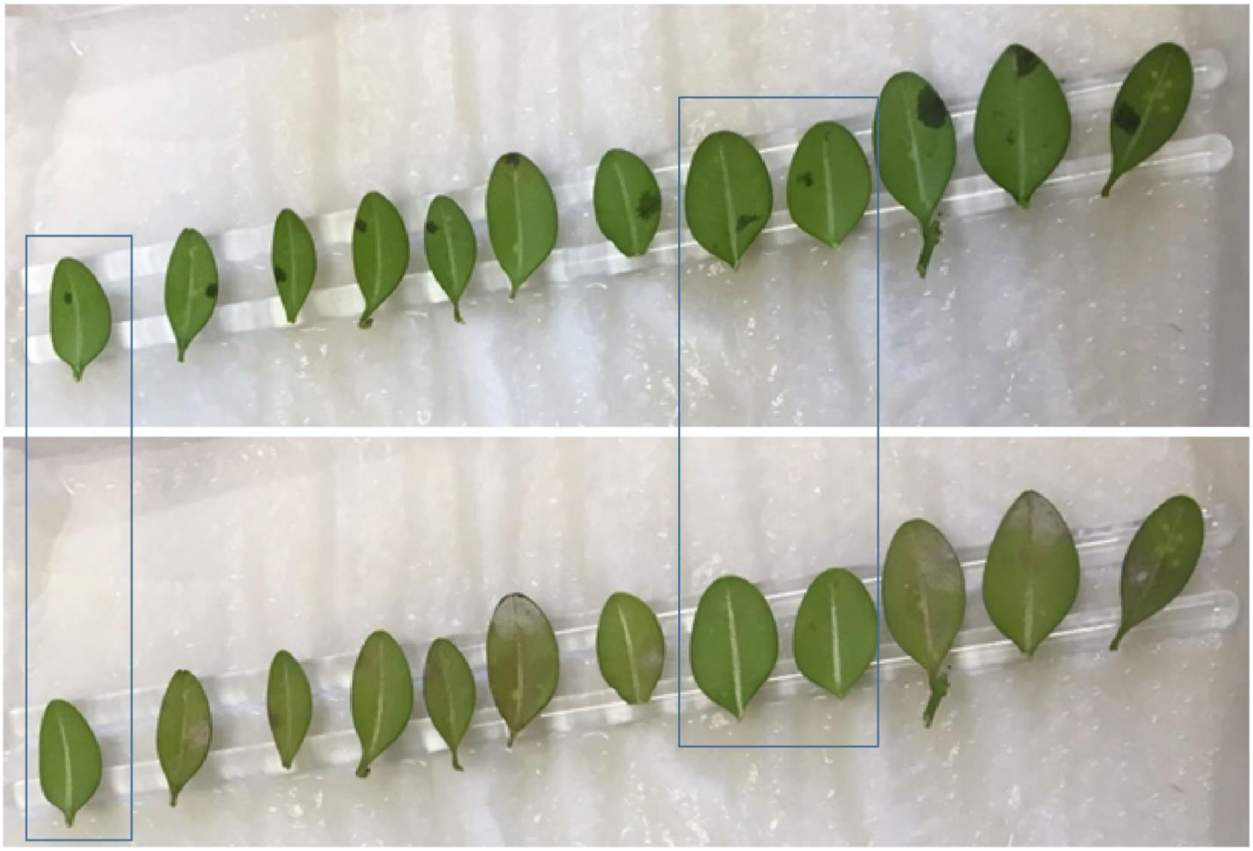

Figure S2: Reduction in colony diameter of 4 week old *Calonectria pseudonaviculata* (*Cps*) by SSG and other boxwood endophytes coded by size (s = small, m = medium, l = large) and color (p = pink, sg = sage green, w = white, y = yellow), plus a number if more than one isolate of similar color types and sizes assessed in three dual culture assays with slightly different *Cps* seeding timings in relation to bacterial streaking: 3 days before (left), at the same time (middle), and 16 h later (right). Each column represents a mean of triplicate plates, topped with a standard error bar. Columns topped with the same letter within each assay did not differ according to the LSD test at $p = 0.05$.

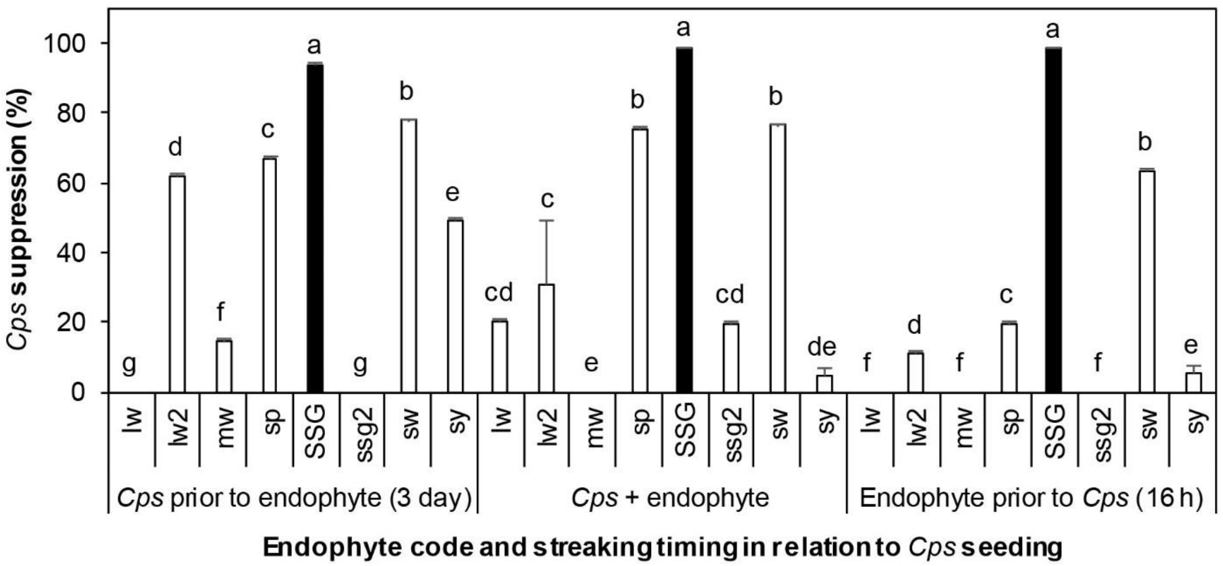

Figure S3: Neighbor-joining tree of SSG (query in yellow) and closely related *Burkholderia* strains in GenBank according to BLAST pairwise alignments of 16S rDNA.

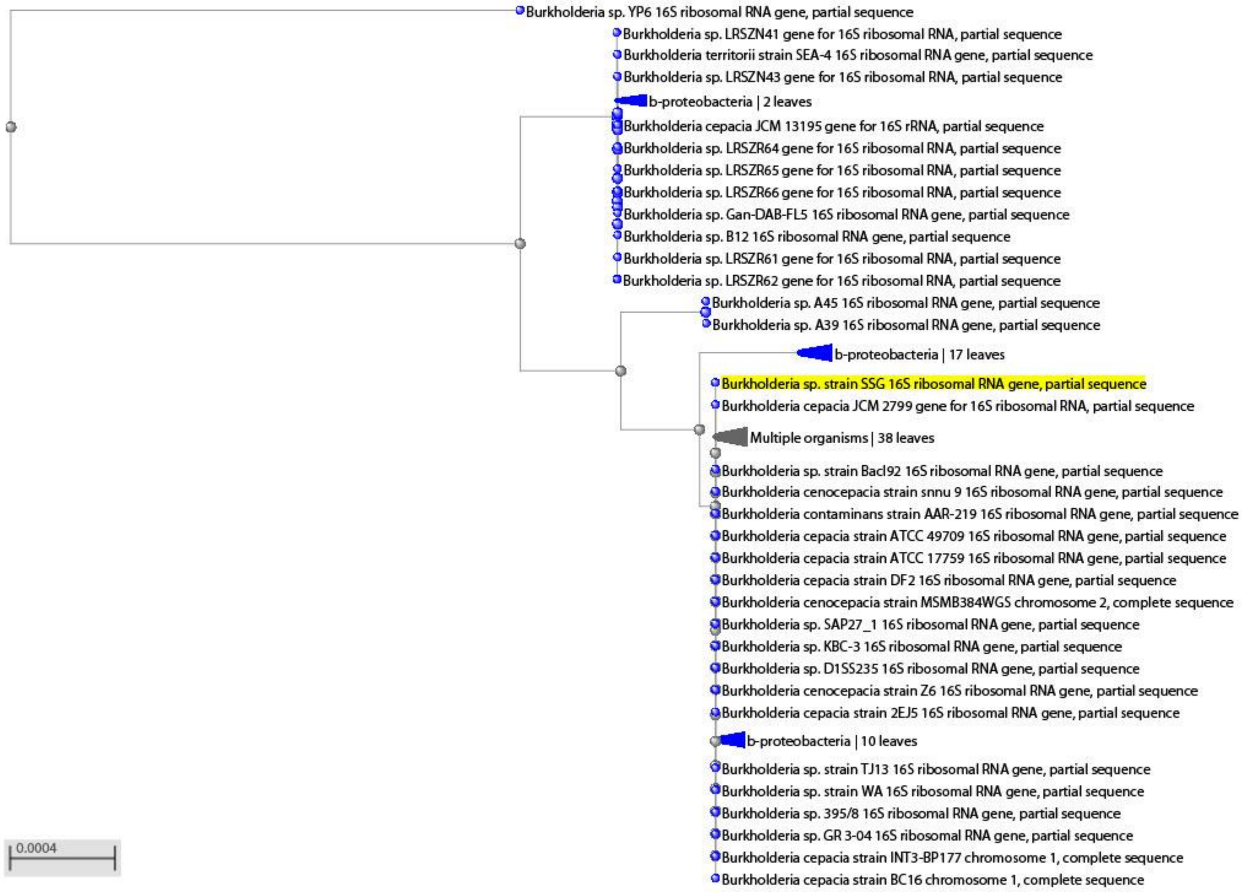

Figure S4: Neighbor-joining tree of SSG (query in yellow) and closely related *Burkholderia* strains in GenBank according to BLAST pairwise alignments of RecA gene.

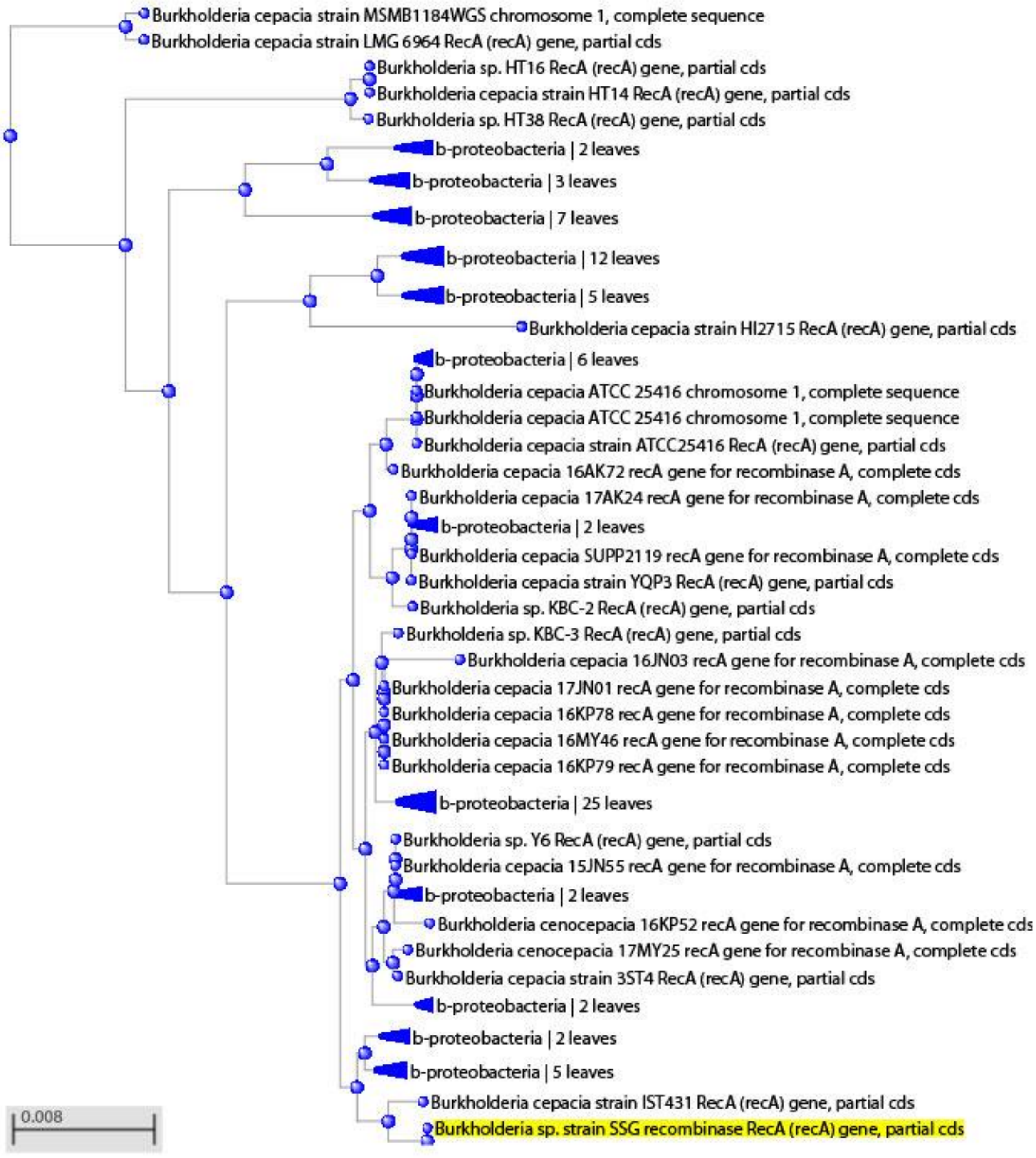

Figure S5: Symptoms on onion scales at 3 days after inoculation with a 10 μl drop of 48 h culture of SSG or nutrient broth (NB) control.

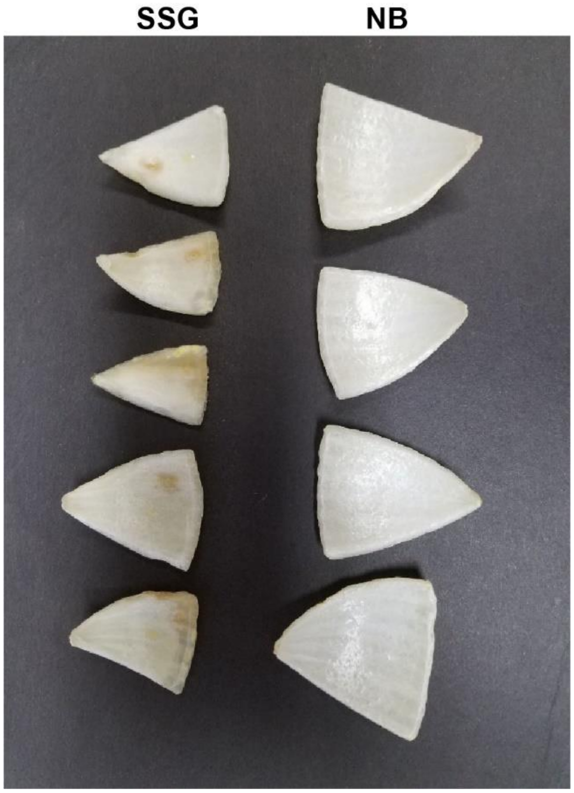

Supplement: Supplementary file 1 [file microorganisms-08-00310-s001.pdf]
